# Statistical elimination of spectral features with large between-run variation enhances quantitative protein-level conclusions in experiments with data-independent spectral acquisition

**DOI:** 10.1186/1471-2105-16-S2-A4

**Published:** 2015-01-28

**Authors:** Lin-Yang Cheng, Yansheng Liu, Ching-Yun Chang, Hannes Röst, Ruedi Aebersold, Olga Vitek

**Affiliations:** 1 Department of Statistics, Purdue University, West Lafayette IN, USA; 2 Department of Biology, Institute of Molecular Systems Biology, ETH Zurich, 8093 Zurich, Switzerland; 3 Faculty of Science, University of Zurich, 8057 Zurich, Switzerland; 4 Department of Computer Science, Purdue University, West Lafayette IN, USA

## Background

Many proteomic investigations summarize the quantitative information across multiple spectral features into protein-level conclusions. Data-independent spectral acquisition (DIA) now generates a lot of interest, as it allows us to quantify many spectral features in a single run. However, the disadvantage of DIA experiments as compared, e.g., to Selected Reaction Monitoring (SRM) is that the features are subject to interferences and noise. We argue that between-run variation provides an additional insight for distinguishing good-quality and noisy DIA features. To appropriately use the quantitative between-run variation, it is important to account for the properties experimental design, and distinguish random artifacts from the biological changes. We have previously proposed a method (Chang et al., ASMS 2013) that accounts for the experimental design to eliminate features with low information content.

## Results

In this project we furthermore emphasized that conducting regularization helps us avoid exploring every subset of features exhaustively, and allows us to conduct hypothesis tests later on so that we would be able to control the false discovery rate of the feature selection process. We evaluated our proposed approach by using three datasets that have some notion of ground truth: an extensive simulation study, a controlled mixture where proteins were spiked into a complex background in known concentrations, and a study of 232 plasma samples, where 18 proteins were quantified in both SWAH and SRM mode in presence of heavy labeled reference peptides. We worked on [1] protein-level estimates of fold changes between conditions, [2] sensitivity and specificity of detecting changes in protein abundance, and [3] accuracy of relative quantification of protein abundance in individual biological samples. A family of linear mixed models similar to that in MSstats http://www.msstats.org were fit to all the datasets. Then we conducted the regularization and hypothesis test to control the selection false discovery rate.

## Conclusion

The results demonstrated that our proposed feature selection approach enhanced sensitivity and specificity of the conclusions, was robust to the amount of noisy fragments, and increased the correlation of subject quantification between SRM and DIA workflows. Importantly, the performance exceeded that of the frequently used 'top 3' approach, which consists of using three spectral features with the highest average intensity between runs. Furthermore, we showed that our proposed approach outperforms using correlation to select the information features.
